# Responses of Water and Salt Parameters to Groundwater Levels for Soil Columns Planted with *Tamarix chinensis*

**DOI:** 10.1371/journal.pone.0145828

**Published:** 2016-01-05

**Authors:** Jiangbao Xia, Ximei Zhao, Yinping Chen, Ying Fang, Ziguo Zhao

**Affiliations:** Binzhou University, Shandong Provincial Key Laboratory of Eco-Environmental Science for Yellow River Delta, Binzhou 256603, China; Hainan University, CHINA

## Abstract

Groundwater is the main water resource for plant growth and development in the saline soil of the Yellow River Delta in China. To investigate the variabilities and distributions of soil water and salt contents at various groundwater level (*G*_L_), soil columns with planting *Tamarix chinensis* Lour were established at six different *G*_L_. The results demonstrated the following: With increasing *G*_L_, the relative soil water content (RWC) declined significantly, whereas the salt content (*S*_C_) and absolute soil solution concentration (*C*_S_) decreased after the initial increase in the different soil profiles. A *G*_L_ of 1.2 m was the turning point for variations in the soil water and salt contents, and it represented the highest *G*_L_ that could maintain the soil surface moist within the soil columns. Both the *S*_C_ and *C*_S_ reached the maximum levels in these different soil profiles at a *G*_L_ of 1.2 m. With the raise of soil depth, the RWC increased significantly, whereas the *S*_C_ increased after an initial decrease. The mean *S*_C_ values reached 0.96% in the top soil layer; however, the rates at which the *C*_S_ and RWC decreased with the *G*_L_ were significantly reduced. The RWC and *S*_C_ presented the greatest variations at the medium (0.9–1.2 m) and shallow water levels (0.6 m) respectively, whereas the *C*_S_ presented the greatest variation at the deep water level (1.5–1.8 m).The RWC, *S*_C_ and *C*_S_ in the soil columns were all closely related to the *G*_L_. However, the correlations among the parameters varied greatly within different soil profiles, and the most accurate predictions of the *G*_L_ were derived from the RWC in the shallow soil layer or the *S*_C_ in the top soil layer. A *G*_L_ at 1.5–1.8 m was moderate for planting *T*. *chinensis* seedlings under saline groundwater conditions.

## Introduction

Shallow groundwater is the primary factor affecting the migration, accumulation and release of soil salt. Differences in the groundwater table can easily lead to the variability of soil water and salt contents, with subsequent effects on the growth, development and distribution of vegetation [1–2]. The relationship between soil water and groundwater transfer is closely related, which is an important focus of investigations into the hydrological cycle and physical soil water processes [3–5]. The hydraulic connection between soil water and groundwater directly influences the water and salt conditions in the soil [6–9]. However, the different objectives, means and methods of researches have led to the independent development of dynamic law for soil water, soil salt, and groundwater in the respective related fields [6,10–12]. Increasing researches into the hydrological cycle have led to a greater awareness of soil water and groundwater and their interconnectedness [13–15]. Groundwater reaches soil layers by capillary upward flow and then may enter the soil water cycle. Research on water cycle processes in the Soil—Plant—Atmosphere continuum should evolve from single-process analyses to comprehensive multi-process analyses to fully understand the migration of soil water and salt as well as groundwater [2–3,14]. For consistency of description, the groundwater table is referred to here as the vertical distance from the soil surface to the phreatic water level (hereafter referred to as *G*_L_). Because of eluviation influences, high salt affinities for soil water [10] and meteorological factors [6], differences in *G*_L_ are the main factor leading to variations in soil water-holding capacities and salt contents in saline soil found in arid inland or muddy coasts that lack freshwater resources [16–18]. Soil water and salt migrations are closely associated with *G*_L_ [10,16,19]. However, with different soil textures [16,20], vegetation types [16,21], micro-topographies [19,22], climatic environments [6,19,23] and other factors, the correlations among the relative soil water content (RWC), salt content (*S*_C_) and *G*_L_ vary greatly within different soil profiles [16,20]. Moreover, the water and salt contents of soil are not completely synchronized change (or have been rising or falling) with the *G*_L_ [18,22,24], and a clear turning point occurs for soil water and salt in response to different *G*_L_ in the soil profile [22,25]. Current research on the water and salt dynamics in soil has mainly focused on the individual relationships between *G*_L_ and soil water or salt [16,20,25–27], and few studies have investigated soil water and salt parameters, such as the RWC, *S*_C_ and absolute soil solution concentration (*C*_S_), in the soil profile and their responses to *G*_L_ along a vertical depth. Presently, the interaction effects and action processes of *G*_L_ with soil water and salt in the soil profile remain unclear, thus leading to difficulties in saline land improvement and water-salt interaction stress, which affects plant growth because of changes in the *G*_L_. Studying the migration characteristics of water and salt in the soil and their interactions with *G*_L_ can help increase the effective prevention against and control of secondary soil salinization at shallow groundwater levels.

Soil salinization is one of the major features of the ecological environment in the Yellow River Delta (YRD). Saline land improvement through vegetation restoration is an important mitigation method among ecological restorations within the muddy coast of the YRD. Because of global climate change, sea level rises and seawater intrusions, the phreatic water level in the saline soil along the muddy coast is generally shallow. Thus, shallow groundwater is a sensitive factor and major water source in the saline soil of the muddy coast of the YRD during the critical period of vegetation growth [1,27–28]. However, the close relationship of soil water and salt with *G*_L_ is a major factor that influences the distribution pattern and community succession of vegetation in the YRD [1,26,28]. The water level and total dissolved solids of groundwater have a “source (groundwater)–sink (soil)” relationship with water and salt in soil [3,5,16,29]. Therefore, the distribution of water and salt in the soil profile at different depths must be investigated to reveal the variability and distribution of soil water and salt at various *G*_L_, and these data will help to fundamentally elucidate groundwater dynamics and soil water and salt variations as well as the associated occurrence of secondary salinization.

*Tamarix chinensis* Lour is a dominant shrub of the YRD, and it can reduce salt, improve soil and water conservation. The salt and water conditions are primary factors influencing the spatial distribution pattern and stand degradation of *T*. *chinensis* vegetation in the YRD [1,28]. Studies have investigated the migration of soil water and salt and its associated relationship with vegetation according to the *G*_L_. Such study has primarily been undertaken in oases of inland saline desert areas [16,20] and agricultural development zones [22,25,30–31], with the research focusing on the water cycle and water use efficiency in arid regions [3,14,25,27]. Research on the relationship between *T*. *chinensis* and *G*_L_ in the YRD is primarily associated with the water and salt conditions on the spatial distribution pattern of *T*. *chinensis* [28] and it’s vegetation ecological effects [1]; however, there is a lack of information on the migration of soil water and salt at various *G*_L_ and their interaction effects.

The objective of this study was to elucidate the distribution and migration of soil water and salt in different soil profiles at various *G*_L_ under saline groundwater conditions as well as in the presence of identical soil textures, plant species and climatic conditions and without surface water sources. Soil columns were established by planting *T*. *chinensis*, a dominant species in the YRD. The *G*_L_ was set to six different levels to simulate saline groundwater conditions in a research greenhouse and analyze the effect of *G*_L_ on variations in the water and salt contents within different soil profiles under *T*. *chinensis* vegetation. Furthermore, this study identified the *G*_L_ required for significant salt accumulation in various soil layers and revealed the dynamic changes that occur for the *G*_L_, soil salt accumulations and soil water levels as well as their coupling effects. The results will provide a reference for the prevention and control of secondary salinization and the efficient use of groundwater resources under the effect of underground saline water.

## Materials and Methods

### Ethics Statement

The research station for this study is owned by Binzhou University. This study was approved by the Research Center of Ecological Environment in Yellow River Delta and the Shandong Provincial Key Laboratory of Eco-Environmental Science for Yellow River Delta.

### Materials

Simulated groundwater was formulated using sea salt from the YRD, which presents a total dissolved solids (TDS) of 20 g·L^-1^, electrical conductivity (EC) of 27.4 ms·cm^-1^, pH of 7.5 and salinity of 1.7%. The experimental soil was collected from the floodplain downstream of the YRD. The soil sample was transported to the laboratory, dried in the air and then crushed, and it was then passed through a 2.0 mm sieve and evenly mixed. The soil sample contained alluvial soil with a field capacity of 37.9%, an initial *S*_C_ of 0.01% and a bulk density of 1.32 g·cm^-3^. Three-year-old *T*. *chinensis* seedlings were uniformly cut to a height of 60 cm before planting, and the average rootstock was 1.3 cm.

### Experimental design

The phreatic water level is relatively shallow in the YRD and generally occurs in a range from 0.5 to 2.5 m [17]. The TDS content of groundwater varies from 14.3 to 32.4 g·L^-1^ [26]. According to the field survey, the *G*_L_ ranges from 0.3 to 2.0 m on the *T*. *chinensis* farm in the Laizhou Bay on the muddy coast of the YRD. Thus, the TDS content of simulated groundwater was set to 20 g·L^-1^, which fell in the range of saline water. The *G*_L_ was set to six water levels, shallow (0.3 m, 0.6 m), medium (0.9 m, 1.2 m) and deep (1.5 m, 1.8 m), with three repetitions at each level.

The experimental design was as follows: Polyvinyl chloride (PVC) pipes of different heights were used as the experimental material to plant *T*. *chinensis* and simulate *G*_L_ in a research greenhouse. The PVC pipes had an inner diameter of 0.30 m, and the height was adjusted according to the simulated *G*_L_. The exact height of the PVC pipes = simulated *G*_L_ + actual flooding depth (0.55 m) + top gap layer above the soil surface (0.03 m). The heights of the PVC pipes were 0.88, 1.18, 1.48, 1.78, 2.08 and 2.38 m, respectively. An inverted layer of quartz sand and a permeable cloth were placed at the bottom of the PVC pipe, to prevent soil leakage from the bottom. Four 1.0 cm water inlet holes were drilled in a row at 10 cm intervals on the PVC pipes from the 0.55 m flooded area and then covered with the permeable cloth. This design allowed for water to enter the soil column from the bottom flooded area and the surrounding inlet holes.

**Fig 1:**
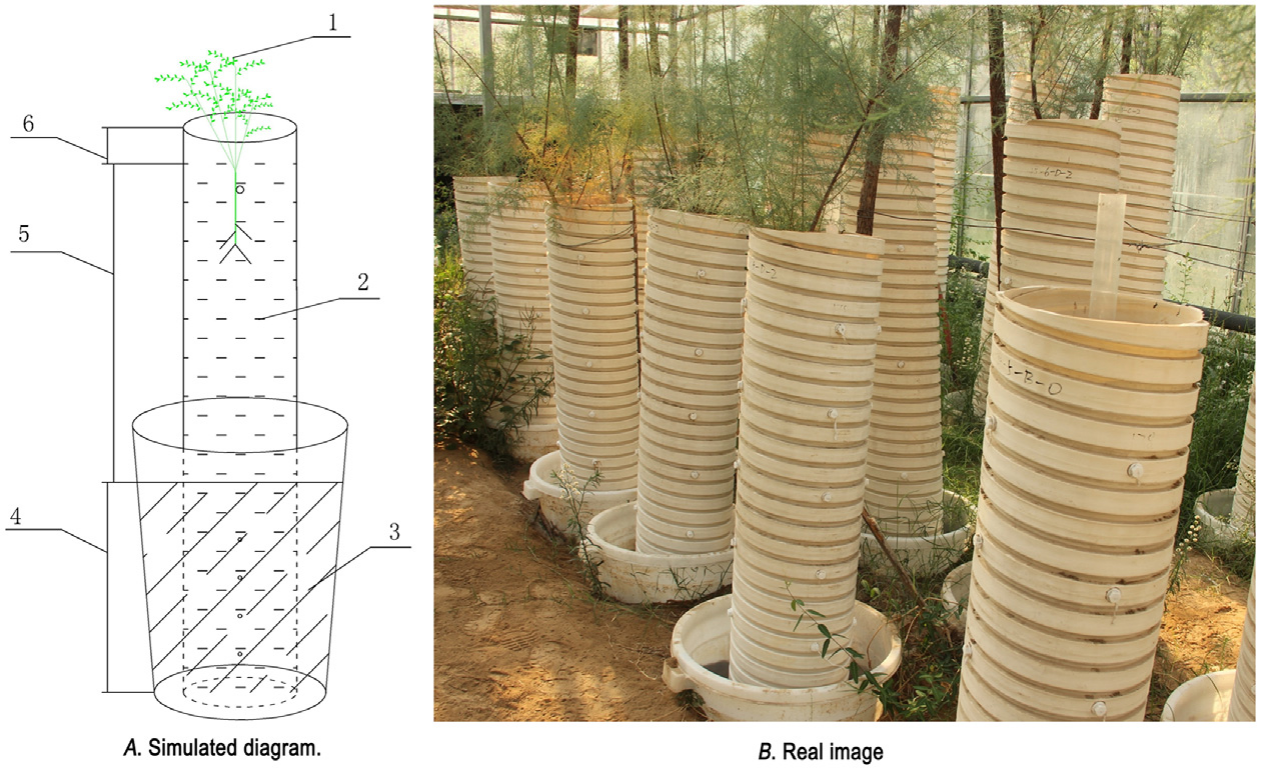
Simulation setup of soil columns planted with *Tamarix chinensis*. 1, *T*. *chinensis*; 2, soil; 3, groundwater; 4, flooded area, 0.55 m; 5, designed groundwater levels, 0.3–1.8 m; 6, gap layer, 0.3 m.

The experiment started on March 3, 2014, and the experimental setup is illustrated in Fig 1. One soil layer was equal to 20.0 cm, and the amount of soil to be loaded was calculated according to the soil bulk density. Air-dried soil was then packed into the PVC pipes, and it was not provided irrigation, fertilization, or other treatments. A trench was then excavated into which a bucket was placed (70.0 cm height; 45.5 cm bottom inner diameter; 57.0 cm top inner diameter), and the bottom of the bucket was isolated from the surrounding soil to ensure the uniformity of the groundwater temperature. PVC pipes containing soil were placed in the large buckets, and then formulated saline water was added to the buckets. The water depth was controlled at 0.55 m, and the soil was supplemented after subsidence occurred in the PVC pipes because of water absorption. The soil columns were allowed to reach an equilibrium and stabilize for 5 days, and then 3-year-old *T*. *chinensis* seedlings were planted in each pipe. Two to three plants were first planted in each container. Fresh water was irrigated from the top of the PVC pipes for all treatments at the early stage of seedling cultivation. Irrigation was performed once every 10 days, with 4.0 L applied each time for a total volume of 12.0 L. Thereafter, surface water was not supplied. Normal cultivation management lasted for 1 month, and 1 seedling was retained from the surviving plants. The groundwater TDS content and the actual immersion depth of the PVC pipes were monitored at 3-day intervals throughout the experimental period, and the groundwater was recharged regularly to maintain a stable water depth and groundwater TDS content. Three months after sowing the *T*. *chinensis* seedlings, soil samples were collected and the water and salt parameters were determined starting on June 5. The simulation design for the soil columns planted with *T*. *chinensis* is shown in Fig 1 (*A*. simulated diagram; and *B*. real image).

### Sample collection and indicator determination

The soil profile sampling interval was designed according to variations in the soil water and salt contents in different soil layers as determined in the simulation experiment in combination with results from the literature [12,22,30]: from the soil surface to a depth of 0–60 cm, a soil layer was equal to 10 cm; from a depth of 60–120 cm, a soil layer was equal to 20 cm; below 120 cm, a soil layer was equal to 30 cm. The top soil layer was collected at a depth of 10 cm for all the soil columns, and soil samples were obtained from soil layers as described above. Three replications were performed for each layer. For convenience of description, the profile of the soil columns was referred to as the top, shallow, medium, deep and bottom layers from top to bottom.

Mass soil water content was determined by the oven-drying method. RWC was calculated as the ratio between mass soil water content and field capacity. *S*_C_ was determined by the residue-drying method with a water/soil ratio of 5: 1. *C*_S_ was calculated as the ratio between *S*_C_ (percentage of dry soil mass) and gravitational soil water content (percentage of dry soil mass). Groundwater salt content, EC and pH were measured using amulti-parameter water quality analyzer in situ (Horiba U52, JPN).

### Data analysis

The experimental data were analyzed using significant difference tests and correlation analyses in Excel 2007 (Microsoft Corp., Redmond, WA, USA) and SPSS 16.0 (SPSS Inc., Chicago, IL, USA).

## Results

### Responses of relative soil water content to different groundwater level

**Fig 2:**
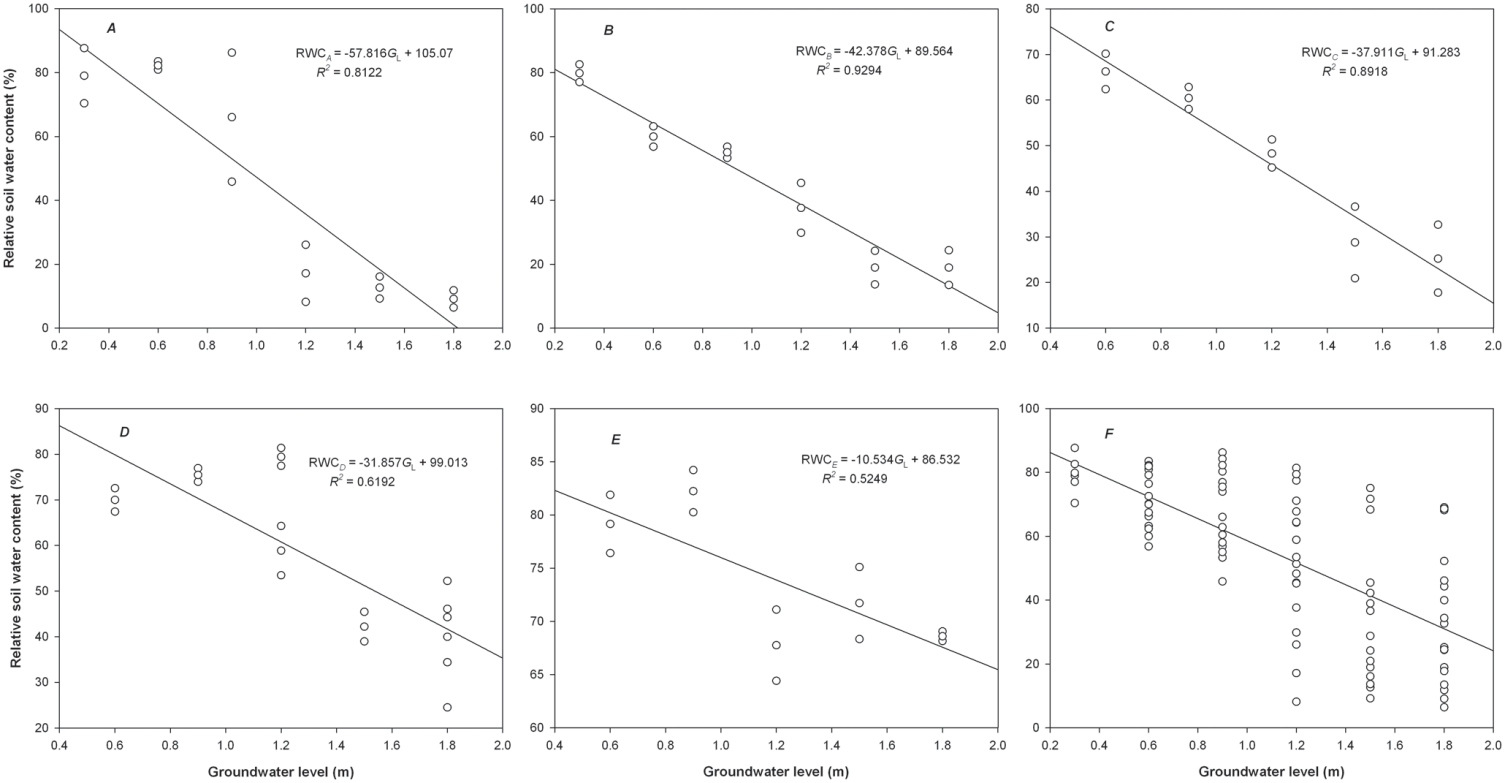
Response of the relative soil water content (RWC) in different soil layers to the groundwater level (*G*_L_). *A*, top soil layer; *B*, shallow soil layer; *C*, medium soil layer; *D*, deep soil layer; *E*, bottom soil layer; *F*, the whole soil columns.

(Fig 2A–2E) shows that the RWC in different soil layers significantly decreased with increases in the *G*_L_ and displayed a negative correlation at the extremely significant level. However, the response relationship of the RWC to *G*_L_ varied greatly with soil depth. To indicate the level of RWC decreases with *G*_L_, the slope (absolute value) of the linear relationship between the RWC and *G*_L_ was descried as the decreasing rate of the RWC with *G*_L_, which was significantly reduced with increasing soil depth. The decreasing rates of the RWC with *G*_L_ in the upper four soil layers (top to deep) were 5.5, 4.0, 3.6 and 3.0 times that in the bottom soil layer. Thus, the decreasing trend in the RWC with increases in the *G*_L_ was gradually diminished towards the deeper soil depths. In the designed *G*_L_ range, the mean RWC presented a relative increase with increasing soil depth, although differences were not observed between the top, shallow and medium soil layers (mean 44.4%–45.9%, *P >* 0.05). However, the deep and bottom soil layers showed a remarkable increase in the RWC, with mean values of 58% and 74%, respectively. The variations in the RWC from the top to the bottom of the soil profile were gradually diminished with increases in the *G*_L_. The RWC occurred in the range of 6%–88% in the 10 cm top soil layer, which presented the greatest variation across the soil profile of 82%. The RWC of the medium soil layer was 18%–70% with 52% variation, and the RWC of the bottom soil layer was 64%–84% with 20% variation. As shown in Fig 2A, the RWC began to decrease significantly in the top soil layer when the *G*_L_ reached 1.2 m. In addition, the soil surface remained wet in all of the soil columns planted with *T*. *chinensis* at *G*_L_ values less than 1.2 m. Thus, the *G*_L_ of 1.2 m was the highest level that groundwater could reach the soil surface and maintain moist conditions.

Fig 2F shows that the mean RWC for the soil profile significantly decreased with increases in the *G*_L_, and the mean RWC at *G*_L_ values of 0.3, 0.6, 0.9, 1.2 and 1.5 m were 2.7, 2.4, 2.3, 1.7 and 1.2 times the mean RWC at a *G*_L_ of 1.8 m (30%), respectively. Variations in the RWC across the vertical profile of the soil columns ranged from 17% to 73% at various *G*_L_, and the RWC first increased and then decreased with increases in the *G*_L_. The RWC presented the greatest variation of 73% at the medium water level with a *G*_L_ of 1.2 m.

### Responses of soil salt content to different groundwater level

**Fig 3:**
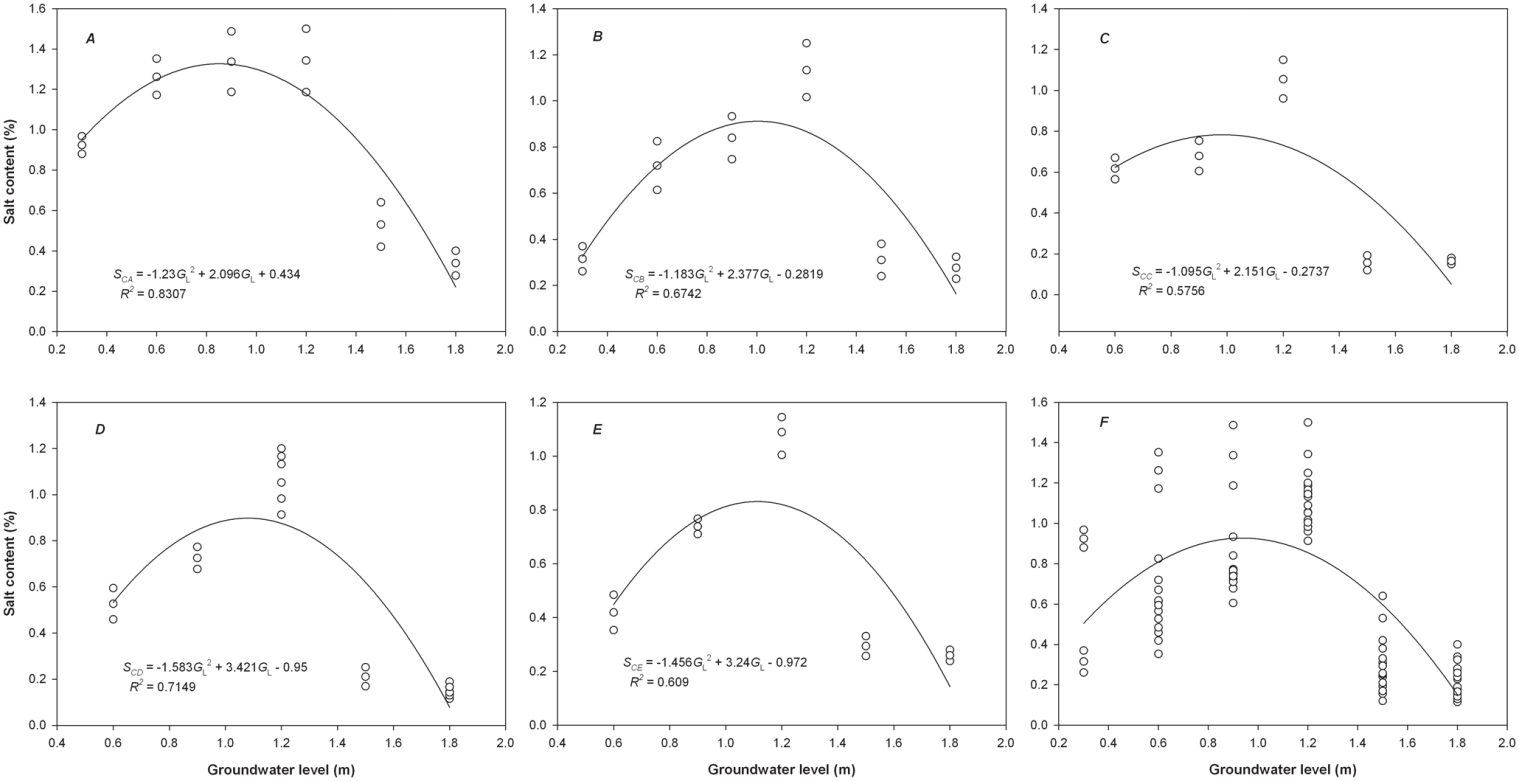
Response of the salt content (*S*_C_) in different soil layers to the groundwater level (*G*_L_). *A*, top soil layer; *B*, shallow soil layer; *C*, medium soil layer; *D*, deep soil layer; *E*, bottom soil layer; *F*, the whole soil columns.

(Fig 3A–3E) illustrates that with an increase in the *G*_L_, the *S*_C_ in different soil layers decreased after initially increasing, with the trend following a parabolic curve. However, with increasing soil depth, the determination coefficient (*R*^2^) of the quadratic function between the *S*_C_ and *G*_L_ increased after initially decreasing, and significant variations in *S*_C_ were observed across the soil profile. Among the different soil profiles, the *S*_C_ was the highest at the medium water level of 1.2 m. Except for the low *S*_C_ in the shallow soil layer at certain *G*_L_ values (shallow 0.3 m; deep 1.5 and 1.8 m) (*P >* 0.05, Fig 3B), the *S*_C_ was significantly higher in the remaining soil layers at the shallow water level compared with the deep water level. In the top soil layer, the *S*_C_ varied from 0.28% to 1.50% in response to various *G*_L_, and it presented a maximum variation of 1.22%. The deep soil layer showed the second greatest variation of the *S*_C_ of 1.09%, and the other soil layers showed minor variations from 0.91%–1.03%. The results showed that the top and bottom soil layers presented greater variability in the *S*_C_ in response to the *G*_L_ compared with the other soil layers.

The mean *S*_C_ in the top soil layer within the range of *G*_L_ (0.3–1.8 m) was obtained according to the integral equation for the fitting function of *S*_C_ and *G*_L_:

$$\bar{S_{\text{C}}}=\frac{1}{1.8-0.3}\int_{0.3}^{1.8}\left(-1.230G_{\text{L}}{}^{\text{2}}+2.096G_{\text{L}}\text{+}0.434\right)dG_{\text{L}}$$

The mean *S*_C_ was 1.10%, which corresponded to a simulated *G*_L_ of 0.42 and 1.28 m. The maximum *S*_C_ was up to 1.33%, which corresponded to a *G*_L_ of 0.85 m. Further analysis revealed that the *S*_C_ was relatively high in the top soil layer at the *G*_L_ of 0.42–1.28 m, and the maximum *S*_C_ as observed at a *G*_L_ of 0.85 m. The mean and maximum values of the *S*_C_ and the corresponding *G*_L_ were obtained for other soil layers using the same method (Table 1). The *G*_L_ values for the *S*_C_ above the mean in the shallow, medium, deep and bottom soil layers were 0.57–1.44 m, 0.57–1.39 m, 0.71–1.45 m and 0.76–1.47 m, respectively. Table 1 shows that within the designed range of the *G*_L_, the mean and maximum values of the *S*_C_ initially increased and then decreased with increasing soil depth. In terms of the mean *S*_C_, the top soil layer was the highest, and it showed strong surface accumulation; the medium soil layer was lower; and the deep and bottom soil layers showed relative increases. The mean values of the *S*_C_ in the top, shallow and deep (bottom) soil layers were 1.8, 1.2 and 1.1 times that in the medium soil layer (0.53%). The maximum values of the *S*_C_ occurred in a *G*_L_ range of 0.78%–1.33%, which corresponded to a theoretical *G*_L_ of 0.85–1.11 m (measured 0.90–1.20 m). The theoretical *G*_L_ values were close to the measured values, indicating that the fitting equation could reflect the quantitative relationship between the *S*_C_ and *G*_L_.

**Table 1:** Groundwater Levels (*G*_L_) for the Mean and Maximum Soil Salt Content (*S*_C_) in Different Soil Layers.

	Mean soil salt content /%		Groundwater level for simulated soil salt mean/m		Groundwater level for soil salt maximum/m		Maximum soil salt content/%	
Soil layer	Measured value	Simulated value	x1	x2	Simulated value	Measured value	Measured value	Simulated value
*A*	0.96	1.10	0.42	1.28	0.85	0.90	1.50	1.33
*B*	0.60	0.69	0.57	1.44	1.00	1.20	1.25	0.91
*C*	0.53	0.60	0.57	1.39	0.98	1.20	1.15	0.78
*D*	0.56	0.69	0.71	1.45	1.08	1.20	1.05	0.90
*E*	0.56	0.65	0.76	1.47	1.11	1.20	1.20	0.83

*A*, top soil layer; *B*, shallow soil layer; *C*, medium soil layer; *D*, deep soil layer; *E*, bottom soil layer.

Fig 3F shows that the mean *S*_C_ for the entire soil profile significantly decreased after initially increasing as the *G*_L_ increased. The highest mean *S*_C_ occurred in soil columns at the medium water level of 1.2 m. Compared with the mean *S*_C_ at a *G*_L_ of 1.2 m (1.12%), the mean *S*_C_ at *G*_L_ values of 0.3, 0.6, 0.9, 1.5 and 1.8 m declined by 43%, 37%, 23%, 73% and 80%, respectively. The variations in *S*_C_ for the entire soil profile ranged from 0.29% to 1.00% at various *G*_L_. As the *G*_L_ increased, the variations in *S*_C_ first increased and then decreased. The greatest variation in the *S*_C_ was 1.00% in the shallow layer with a water level of 0.6 m, and the lowest variation in the *S*_C_ was 0.29% in the deep layer with a water level of 1.8 m.

### Responses of absolute concentration of soil solution to different groundwater level

**Fig 4:**
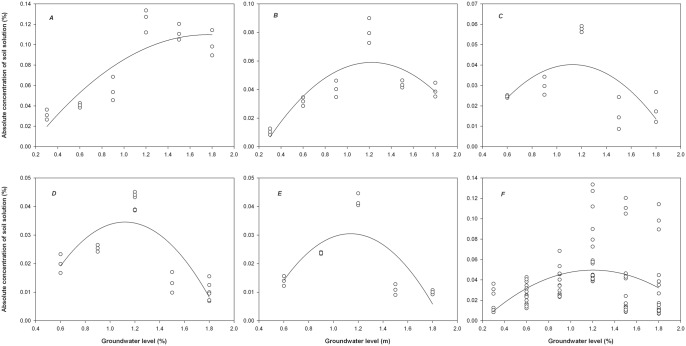
Response of the absolute concentration of soil solution (*C*_S_) in different soil layers to the groundwater level (*G*_L_). *A*, top soil layer; *B*, shallow soil layer; *C*, medium soil layer; *D*, deep soil layer; *E*, bottom soil layer; *F*, the whole soil columns.

(Fig 4A–4E) shows that with increases in the *G*_L_, the *C*_S_ followed an initial increasing and then decreasing trend in different soil layers, although the top soil layer mainly exhibited an increasing trend. The response relationship of the *C*_S_ with *G*_L_ varied substantially with soil depth. Throughout the soil profile, the *C*_S_ was the highest at the medium water level of 1.2 m. However, the top and shallow soil layers had significantly higher *C*_S_ at the deep water level compared with the shallow water level, whereas the other soil layers showed higher *C*_S_ at the shallow and medium water levels compared with the deep water level. The *C*_S_ variations in the different soil layers ranged from 0.04% to 0.11%. As the soil depth increased, the *C*_S_ variations decreased with increases in the *G*_L_. The greatest variations occurred in the top soil layer and then the shallow soil layer, and significant differences were not observed in the middle, deep, or bottom soil layers (*P >* 0.05). The mean *C*_S_ declined significantly with increasing soil depth. The deep and bottom soil layers had the same mean *C*_S_ of 0.02%, whereas the top, shallow and medium soil layers were 4.0, 2.0 and 1.5 times the value of the deep (bottom) soil layer.

Fig 4F shows that throughout the soil profile, the mean *C*_S_ significantly decreased after initially increasing with increases in the *G*_L_. The highest mean *C*_S_ of 0.06% occurred at the medium water level of 1.2 m, whereas the mean *C*_S_ values at water levels of 0.3, 0.6, 0.9, 1.5 and 1.8 m decreased by 67%, 50%, 33%, 33% and 50%, respectively. The *C*_S_ variations throughout the soil profile were 0.03%–0.11% at various *G*_L_. An upward trend in *C*_S_ variations was observed with increases in the *G*_L_ values. The *C*_S_ was relatively stable at the shallow water level but fluctuated greatly at the medium and deep water levels.

### Correlations between soil water and salt parameters and groundwater level

Table 2 shows the Pearson correlation coefficients (PCC) between the soil water and salt parameters and *G*_L_ in different profiles. The correlations between the RWC, *S*_C_, *C*_S_ and *G*_L_ showed substantial differences with soil depth. The RWC presented extremely significant negative correlations with *G*_L_ in the various profiles, and the PCC values first increased and then decreased with increasing soil depth. The negative correlation between the RWC and *G*_L_ was most significant in the shallow soil layer, indicating that the measured RWC could be used to predict the *G*_L_ in this soil layer, and the prediction function is expressed as follows:

$$\text{RWC }=\;-42.37G_{\text{L}}\;+\;89.564m(R^{2}\;=\;0.9294)$$

**Table 2:** Correlation Coefficients between the Soil Water and Salt Parameters and Groundwater Level (*G*_L_) Values.

Soil layer	Soil water and salt parameters	Groundwater level	Relative soil water content	Soil salt content
*A*	RWC	-0.901**		
	*S*_C_	-0.611**	0.578**	
	*C*_S_	0.438	-0.611**	0.025
*B*	RWC	-0.965**		
	*S*_C_	-0.166	-0.163	
	*C*_S_	-0.509*	-0.561*	0.666**
*C*	RWC	-0.943**		
	*S*_C_	-0.589*	0.650**	
	*C*_S_	-0.234	0.258	0.987**
*D*	RWC	-0.791**		
	*S*_C_	-0.558**	0.792**	
	*C*_S_	-0.458*	0.593**	0.950**
*E*	RWC	-0.715**		
	*S*_C_	-0.342	0.013	
	*C*_S_	-0.243	-0.178	0.985**

*indicates significant correlation at *P* < 0.05;

**indicate significant correlation at *P* < 0.01.

RWC, relative soil water content; *S*_C_, soil salt content; *C*_S_, soil solution absolute concentration; *A*, top soil layer; *B*, shallow soil layer; *C*, medium soil layer; *D*, deep soil layer; *E*, bottom soil layer.

The accuracy of the *G*_L_ estimations was the highest using the RWC in the shallow soil layer, which was followed by the RWC of the medium and top soil layers, and the accuracy was the lowest using the RWC measured in the deep and bottom soil layers. The analysis revealed that the *G*_L_ had a greater impact on the RWC in the top, shallow and medium soil layers and then the deep soil layer. The lowest impact was observed in the bottom soil layer adjacent to the groundwater.

The PCC values of the *S*_C_ and *G*_L_ show that the *S*_C_ in the top soil layer had the most significant negative linear correlation with *G*_L_, and it was followed by the *S*_C_ in the deep and medium soil layers; however, the shallow and bottom soil layers did not show a correlation between the two factors. Soil evaporation may have promoted the upward migration of salt in the groundwater and weakened the link between the groundwater and the middle soil layer, thereby reducing or slowing down the groundwater's impact on the accumulation of soil salt. Sun et al. found a similar trend between the *G*_L_ and soil salt accumulation [30]. A correlation in this study was not observed between the *G*_L_ and *S*_C_ in the shallow or bottom soil layer, which may have been because the bottom layer was adjacent to the water level and the soil water was close to saturation, thus accounting for the relatively stable *S*_C_. In addition, the shallow soil layer might have been associated with substantial adsorption of soil salt by the roots of *T*. *chinensis*. The PCC values of the *C*_S_ and *G*_L_ show that the *C*_S_ in the shallow and deep soil layers was negatively correlated with the *G*_L_, whereas correlations were not observed in the other two soil layers.

With regard to the interaction effects between the soil water and salt parameters, the *C*_S_ was in negatively correlated with the RWC at the highly significant (*P* < 0.01) and significant levels (*P* < 0.05) in the top and shallow soil layers, respectively, whereas a highly significant positive correlation (*P* < 0.01) was observed in the deep soil layer. The RWC and *S*_C_ were positively correlated at the highly significant level in the surface, medium and deep soil layers, although correlations were not observed in the other soil layers. Except for the top soil layer, the *C*_S_ and *S*_C_ were positively correlated at the highly significant level in the remaining soil layers. Throughout the soil profile, the *S*_C_ and RWC were negatively correlated with the *G*_L_, whereas the *S*_C_ and RWC were positively correlated with each other at the significant level. With increases in the *G*_L_, the RWC and *S*_C_ throughout the soil columns both declined. Because the *C*_S_ was jointly affected by mass soil water content and *S*_C_, it exhibited limited correlations with the *G*_L_.

## Discussion

### Coupling effect between relative soil water content and groundwater level

Groundwater and soil water have a close hydraulic connection because groundwater supplies water to the soil through the inherent water potential of the vadose zone or plant transpiration [17]. The present study showed that with increasing soil depth, the mean RWC displayed an upward trend at various *G*_L_; however, the rate at which the RWC decreased with the *G*_L_ was significantly diminished. The RWC of soil columns decreased with increases in the *G*_L_, with a highly significant negative correlation occurring between the two parameters. This result is similar to findings in the literature, which reported a significant negative correlation between mass soil water content and *G*_L_ in a desert oasis [20] and shallow groundwater areas [3,12]. When the *G*_L_ is shallower, capillary water formed at the water level is brought close to the soil surface. With relatively low atmospheric humidity, water at the capillary meniscus can change from the liquid phase to gaseous phase and directly enter the atmosphere. Groundwater will continue to migrate and evaporate by capillarity, resulting in higher soil water content in the shallow groundwater area [3,27,32]. As the *G*_L_ increases, the path of groundwater migration upward is extended to reach the top and shallow soil layers. In addition, the water-transporting capacity of the vadose zone is diminished and the water EC is reduced, thus decreasing groundwater recharge of the soil water [24]. The soil water content in the top or shallow soil layers decreases and a dried layer is formed; therefore, the RWC for this soil profile significantly decreased with increases in the *G*_L_. For different soil layers, the correlation between the RWC and *G*_L_ first increased and then decreased with increasing soil depth (Table 2) because the soil profiles closer to the groundwater experienced a stronger water-transporting capacity in the vadose zone, and the variability in the RWC was reduced. At a *G*_L_ of less than 0.9 m, the RWC occurred at higher levels in the top soil layer and showed minor variations in the vertical profile. This trend could be related to the dual effects of atmospheric evaporation and capillary transportation of water in the surface soil at the shallow water level [33]. When the *G*_L_ exceeded 1.2 m, the variations in the top RWC were diminished, which was mainly because of the reduced groundwater recharge to the surface soil water with increases in the *G*_L_. In addition, when the water level exceeds the critical depth for capillarity, a water-deficient dried soil layer is formed at the soil surface. Research has shown that the *G*_L_ could significantly alter certain soil water parameters, such as the mass soil water content, soil water deficit and gravity capacity in water reservoirs [5,27]. The soil water exhibited an increasing trend from top to bottom along the vertical profile in the Keriya Oasis. In addition, the relationship between the RWC and *G*_L_ gradually enhances with the adding soil depth, and the strongest negative correlation has been shown to occur at a depth of 15–20 cm [20]. In the present study, the strongest negative correlation between the RWC and *G*_L_ was found in the 20–40 cm shallow soil layer. However, other studies have shown that at *G*_L_ > 1.0 m, the soil water in the 0–10 cm top soil layer is not correlated with the *G*_L_ [12]. Therefore, a specific water level threshold may occur for the impact of *G*_L_ on the RWC in the soil profile, and the *G*_L_ of 1.2 m may be the critical depth for groundwater migration along the soil columns. The RWC presented the greatest variations at the *G*_L_ of 1.2 m, and a remarkable decrease occurred in the top RWC at *G*_L_ values that exceeded 1.2 m.

### Coupling effect between soil salt content and groundwater level

As a carrier of salt, groundwater directly affects the soil salt variations. Thus, salt migration with water is the primary pathway of soil salt migration. Water migration leads to the migration and accumulation of soil salt [8,17], and soil salt accumulation because of phreatic water evaporation generally corresponds to a reduction in the *G*_L_ and lasts until the *G*_L_ drops below a critical depth [24]. When the *G*_L_ exceeds the depth limit for evaporation [3], the groundwater cannot easily migrate to the shallow soil layer. Research has found that at shallower *G*_L_, the *S*_C_ decreases with increases in the *G*_L_, which conforms to a negative correlation [24,33–34] or an exponential relationship [14,22,25]. Deng et al. [8] found that the coupling coefficient between the *G*_L_ and *S*_C_ remained above 0.7 in the Keriya Oasis. However, these two factors are not synchronous with an increase or decrease [12]. When the *G*_L_ reaches a certain depth, the soil EC tends to reach a constant value that is proportional to the *G*_L_ [22]. The present study showed that with increases in the *G*_L_, the *S*_C_ first increased and then decreased in various soil profiles. However, the highest *S*_C_ always occurred in the top soil layer regardless of the *G*_L_, and a clear phenomenon of surface accumulation occurred. The effect of salt accumulation in the top soil layer varied with different *G*_L_. Yao and Yang [26] also found that the *S*_C_ profiles were characterized by surface and bottom accumulations in the YRD.

In the same soil profile, the *S*_C_ and *G*_L_ did not present a single linear correlation (Fig 3, Table 2), which was primarily because of the interactive and mutually influencing internal self-adaptive and self-regulating process that occurs between groundwater and soil water in common fields [12,24]. In the present study, the simulation experiment was conducted under the conditions of stable TDS and *G*_L_; thus, sufficient groundwater and salt were available to supplement losses of water and salt because of phreatic water evaporation. However, using the medium water level of 1.2 m as the boundary, at *G*_L_ < 1.2 m, the *S*_C_ of different soil profiles declined with decreasing *G*_L_. The soil water content at lower *G*_L_ values may have been relatively high, and the soil salt accumulated in the surface layer may have increased the osmotic pressure and reduced the evaporation rate [10,14], thus resulting in a downward trend in *S*_C_. At *G*_L_ > 1.2 m, the *S*_C_ of different soil profiles declined with increases in the *G*_L_. These two factors conformed to a negative correlation (right side of a parabolic trend line). This result is consistent with that of relevant studies [24,33–34]. The varying trends of the *S*_C_ with the *G*_L_ in the soil columns may have been caused by the different patterns of active salt accumulation in the soil surface and within the soil body [13,29]. When the *G*_L_ is less than the critical depth for phreatic water evaporation, the groundwater mainly reaches the soil surface by capillary action, and membranous water also reaches the soil surface, thus leading to active salt accumulation. When the *G*_L_ is relatively increased, the soil salt rapidly accumulates in the surface layer to form a crust-like protective layer that reduces evaporation and results in a decreasing trend in the *S*_C_. When the *G*_L_ exceeds the critical depth for phreatic water evaporation, salt cannot reach the soil surface, and a portion of the salt accumulates in the soil to form residual saline soil [13,29].

In the soil columns planted with *T*. *chinensis*, the greatest *S*_C_ at a *G*_L_ of 1.2 m was 1.2 and 3.1 times the *S*_C_ at a *G*_L_ of 0.3 and 1.8 m, respectively, whereas the bottom *S*_C_ at a *G*_L_ of 1.2 m was 2.6 and 4.2 times that of the *G*_L_ at 0.6 and 1.8 m, respectively. Similarly, Chen et al. found that the salt accumulation rate in the surface soil exhibited a clear decreasing trend with increases in the *G*_L_ in the Kashgar area of the Xinjiang Province, China, and they showed that the salt accumulation at a *G*_L_ of 25 cm was more than 2 times that at a *G*_L_ of 50 cm [33]. Our analysis showed that the maximum *S*_C_ in the various soil profiles corresponded to a specific *G*_L_. A threshold *G*_L_ may occur for *S*_C_ variations with *G*_L_, and 1.2 m is likely the critical *G*_L_ for shifts in soil salt accumulation because at this *G*_L_, the soil salt accumulation was the highest in the soil columns.

In the soil columns planted with *T*. *chinensis*, the *S*_C_ presented the greatest variations in the 0–10 cm top soil layer and then the deep soil layer, whereas the other soil layers showed minor differences. This result is similar to results observed for the salinization irrigation area in Hetao, Inner Mongolia, in which the soil EC showed maximum variations with increases in the *G*_L_ in the 0–20 cm surface soil layer [22]. With increasing soil depth, the coefficient of determination (*R*^2^) for the quadratic function of the *S*_C_ and *G*_L_ first declined and then increased, whereas the corresponding PCC values showed great variability. Li et al. found that the exponential correlation between the soil EC and *G*_L_ was reduced by layers with increases in the *G*_L_ in the salinization irrigation area of Hetao, Inner Mongolia, and the strongest correlation between the EC and *G*_L_ was obtained in the surface soil layer [22]. Sun et al. found that the *S*_C_ was negatively correlated with the *G*_L_ for the plow layer and bottom layer in the agricultural development zone in Karamay, whereas significant correlations were not found for the middle layer [30]. In addition to its close relationship with *G*_L_, the degree of salt accumulation in various soil profiles is also associated with the vegetation type [5,21], meteorological factors [6,8,22] and hydrogeological and topographical conditions [16–17,20]. However, the *G*_L_ is a decisive condition for the occurrence of soil salinization [8,11,35]. To prevent regional soil salinization, reasonable practices should be implemented to control the *G*_L_ at a depth that will not lead to salt accumulation in the soil through evaporation. Combined with the *G*_L_ for relatively high *S*_C_ in the top and shallow soil layers (Table 1), the moderate *G*_L_ for *T*. *chinensis* seedling growth should be greater than 1.2 m, and *G*_L_ values in the range of 1.5–1.8 m are preferable.

Waterlogging, water deficits, or high *S*_C_ values can affect plant growth. The *C*_S_ is an important parameter for characterizing the relationship of plant growth with soil water and salt, and it can be used to represent the soil water and salt conditions required for plant growth. In this study, the highest *C*_S_ occurred in the top soil layer of the soil columns, and the values significantly decreased with increasing soil depth. The downward trend of the *C*_S_ with soil depth could be related to the higher *S*_C_ and lower RWC in the upper soil layers and the lower *S*_C_ and higher RWC in the bottom soil layer.

## Conclusions

With increasing *G*_L_, the RWC significantly decreased in different soil profiles and throughout the entire soil column planted with *T*. *chinensis*, whereas variations in the RWC first increased and then decreased. Throughout the soil profile, the RWC presented the greatest variations in the middle water level at 0.9–1.2 m and the most stable variations in the shallow water level at 0.3–0.6 m. The RWC variations from the top to the bottom of the soil profile were gradually reduced with increasing *G*_L_, and as the *G*_L_ neared the shallow and top soil layers, more dramatic RWC variations were observed.

With increasing *G*_L_, the *S*_C_ and *C*_S_ first increased and then decreased in different soil layers. A *G*_L_ of 1.2 m was the threshold for variations in the soil water and salt because at this level, the *S*_C_ and *C*_S_ reached the highest levels in various soil profiles. A *G*_L_ of 1.2 m was also the highest level at which the groundwater could keep the soil surface moist in the soil columns. Throughout the soil profile, the *S*_C_ varied most dramatically at the shallow water level and then the middle water level, and it was most stable at the deep water level. The mean values of the *S*_C_ first decreased and then increased, whereas the *C*_S_ displayed a significant downward trend with increasing soil depth.

The RWC, *S*_C_ and *C*_S_ were closely related to the *G*_L_ in the soil columns. However, the correlations between the various parameters showed significant differences in the various soil profiles. The RWC and *S*_C_ had the strongest negative correlation with the *G*_L_ in the shallow and top soil layers. The shallow RWC and top *S*_C_ could be used to predict the *G*_L_ because the highest accuracy of *G*_L_ estimation was observed in these layers. *T*. *chinensis* seedlings should primarily be planted in the shallow soil layer, and the most moderate *G*_L_ is from 1.5 to 1.8 m.
